# Update on Schnelle et al and Expression of Editorial Concern

**DOI:** 10.2196/16313

**Published:** 2019-09-23

**Authors:** JMIR Editorial Office

## Background

The Office of the Deputy Vice-Chancellor at the University of Queensland (UQ) has concluded its research misconduct investigation regarding the two papers, hereafter referred to as Paper 1 (the protocol in JMIR Research Protocols) [1] and Paper 2 (the results in JMIR Public Health and Surveillance) [2]. In short, the main findings were that no retraction is required.

There were issues with inadequate disclosure of conflict of interests on submission, as well as “honest” mistakes, both of which were fully addressed in the JMIR Expression of Editorial Concern, Correction of Conflict of Interest and Affiliation, and Data Corrections, which we published in both affected journals [3,4] in close consultation with the Committee on Publication Ethics (COPE), together with the updated conflict of interest statement and data corrections submitted by the authors. According to the Council of Science Editors (CSE), the purpose of an Expression of Editorial Concern is to draw attention to potential problems in a publication, “but it does not go so far as to retract or correct an article.” CSE continues that “an editor who has a significant concern about the reliability of an article but not enough information to warrant a retraction until an institutional investigation is complete will sometimes use an expression of concern” [5]. COPE’s guidelines encourage editors to consider an Expression of Editorial Concern if “they receive inconclusive evidence of research or publication misconduct by the authors” or if “an investigation is underway but a judgement will not be available for a considerable time” [6], which both fit the circumstances of this case.

## Response

As the University of Queensland investigation is now complete, we are updating the Expression of Editorial Concern following the key findings of the university report, from which we cite below, together with our response, as follows:

**University of Queensland:**

The investigation established that a number of authors of the above-named publications have associations with Universal Medicine. The investigation found that the conflict of interest statements submitted with the papers, which were not subsequently published by JMIR, indicate that there is a potential for a conflict of interest but do not adequately declare the nature of the authors’ associations with Universal Medicine. Those associations were subsequently adequately detailed in the updated conflict of interest statement by the authors published in the JMIR Expression of Editorial Concern, Correction of Conflict of Interest and Affiliation, and Data Corrections (doi:10.2196/publichealth.9932).

**JMIR response:** The investigation confirms our concerns that the vaguely phrased originally submitted COI (disclosing that the authors are “insiders”) did “not adequately declare the nature of the authors’ associations with Universal Medicine”. No changes to the Expressions of Editorial Concern or corrections are deemed necessary because they point out exactly that concern. JMIR may not have accepted the papers if the editor and reviewers had been aware of the full extent of the authors’ COIs, or at least would have insisted on describing ways to manage the significant COIs of the authors.

**University of Queensland:**

In relation to the concern about statistical errors in Paper 2, the authors submitted a correction to JMIR prior to the University becoming aware of the concerns. In this instance the authors acted to correct the error as soon as possible after becoming aware of it. There is no evidence that these were more than honest mistakes. The errors were corrected in the JMIR Expression of Editorial Concern, Correction of Conflict of Interest and Affiliation, and Data Corrections (doi:10.2196/publichealth.9932)”

**JMIR response:** To reflect the UQ judgement that “there is no evidence that these were more than honest mistakes” we have updated the Expressions of Editorial Concern [3,4] to remove the following paragraph:

Finally, we are very concerned that the original results paper contained large statistical errors inflating the effect sizes (now corrected, see data correction below), which the authors themselves corrected (see below). We are giving the authors the benefit of the doubt in assuming that these were honest mistakes and not intentional errors, but it casts further doubts on the level of oversight as well as vetting of the data by an independent person.

**University of Queensland:**

The investigation determined that there are errors in the language used to describe the status of the study in Paper 1. Specifically, Paper 1 describes a planned study as if it had not begun, but at the time of publication data collection had been completed. The investigation found that the inaccuracy in reporting of the study status represents an inadvertent error. Nonetheless, the language used may give an inaccurate impression of the study status that requires correcting.

**JMIR response:** We have inserted the phrase “At the time of publication of this protocol, data collection has been completed” in the Methods section of the abstract and body of the protocol paper (Paper 1 [1]). In addition, we have added an IRRID (International Registered Report Identifier) to both papers [1,2], which indicate (through the prefix DE for “Data Existing”) that the data were already collected at the time as is now standard practice for all protocols we publish [7].

The correction will appear in the online version of the papers on the JMIR website on September 23, 2019, together with the publication of this correction notice. Because this was made after submission to PubMed, PubMed Central, and other full-text repositories, the corrected articles have also been resubmitted to those repositories.

## Abbreviations

CSE: Council of Science Editors
COPE: Committee on Publication Ethics
UQ: University of Queensland

## References

[1] Schnelle C, Minford Ej, McHardy V, Keep J (2017). A Group of 500 Women Whose Health May Depart Notably From the Norm: Protocol for a Cross-Sectional Survey. JMIR Res Protoc.

[2] Schnelle C, Minford Ej, McHardy V, Keep J (2018). Comparative Analysis of Women With Notable Subjective Health Indicators Compared With Participants in the Australian Longitudinal Study on Women’s Health: Cross-Sectional Survey. JMIR Public Health Surveill.

[3] JMIR Editorial Office (2018). Expression of Editorial Concern, Correction of Conflict of Interest and Affiliation. JMIR Res Protoc.

[4] JMIR Editorial Office (2018). Expression of Editorial Concern, Correction of Conflict of Interest and Affiliation, and Data Corrections. JMIR Public Health Surveill.

[5] Scott-Lichter D, Editorial Policy Committee, Council of Science Editors (CSE) (2012). CSE’s white paper on promoting integrity in scientific journal publications.

[6] Wager E, Barbour V, Yentis S, Kleinert S, Committee on Publication Ethics (COPE) (2019). Retraction guidelines.

[7] JMIR Publications What is an International Registered Report Identifier (IRRID)?.

